# Incidence and clinical relevance of persistent iatrogenic atrial septal defect after percutaneous mitral valve repair

**DOI:** 10.1038/s41598-021-92255-3

**Published:** 2021-06-16

**Authors:** Mhd Nawar Alachkar, Anas Alnaimi, Sebastain Reith, Ertunc Altiok, Jörg Schröder, Nikolaus Marx, Mohammad Almalla

**Affiliations:** Department of Cardiology, Angiology and Intensive Care, Medical Clinic I, University Hospital RWTH Aachen, Pauwelsstrasse 30, 52074 Aachen, Germany

**Keywords:** Cardiology, Interventional cardiology

## Abstract

Percutaneous mitral valve repair (PMVR) requires transseptal puncture and results in iatrogenic atrial septal defect (iASD). The impact of persistent iASD was previously investigated. However, data were diverse and inconclusive. 53 patients who underwent MITRACLIP were retrospectively included. Based on the presence of iASD in transesophageal echocardiography (TEE) after 6 months, patients were divided in two groups (iASD group vs. non-iASD group). Impact of iASD on outcome at 6 months and at two years was evaluated. Persistent iASD was detected in 62% of patients. Independent predictors for persistent iASD were female gender and reduced left ventricular ejection fraction. At 6-month follow-up, there was no difference in reduction of NYHA class (ΔNYHA = 1.3 ± 1 in iASD group vs. 0.9 ± 1 in non-iASD group, p = 0.171). There was a significant difference in right ventricular end diastolic diameter (RVEDd) (42 ± 8 mm in iASD-group vs. 39 ± 4 mm in non-iASD group, p = 0.047). However, right ventricular systolic function (TAPSE) (14 ± 7 mm in iASD group vs. 16 ± 8 mm in non-iASD group, p = 0.176) and right ventricular systolic pressure (RVSP) (40 ± 12 mmHg in iASD group vs. 35 ± 10 mmHg in non-iASD group, p = 0.136) were still comparable between both groups. At 2 years follow-up, there was no significant difference regarding rate of rehospitalization (24% vs 15%, p = 0.425) or mortality (12% vs 10%, p = 0.941) between both groups. Incidence of persistent iASD after MITRACLIP is markedly high. Despite the increase in right ventricular diameter in patients with persistent iASD, these patients were not clinically compromised compared to patients without persistent iASD.

## Introduction

Percutaneous mitral valve repair (PMVR) with MITRACLIP is a well-established therapeutic option for treatment of severe mitral regurgitation (MR) in patients with high surgical risk^1–3^. MITRACLIP procedure requires transseptal puncture and results in an iatrogenic atrial septal defect (iASD) which may persist after the procedure^4^. The prevalence of persistent iASD was previously investigated^5–8^. The clinical relevance of persistent iASD was evaluated in two prospective studies^9,10^. However, the results of both studies were diverse. In one study, persistent iASD was associated with a worse prognosis^9^. In the other one, closure of iASD after PMVR was not associated with any improvement in the clinical outcome, compared to conservative therapy^10^. In summary, available data about the impact of persistent iASD on clinical and echocardiographic outcomes is still scarce and inconclusive^11^. This study sought to evaluate the incidence and the impact of persistent iASD on clinical and echocardiographic outcomes after PMVR. Furthermore, predictors for persistent iASD were investigated.

## Methods

### Patients’ inclusion

We screened our register of all patients who underwent MITRACLIP procedure from January 2014 till December 2015 (n = 154). Patients who presented to the predetermined elective follow-up appointment at our center with clinical interview and echocardiographic examinations with transesophageal echocardiography (TEE) at 6 months were retrospectively included (n = 53). Patients who received their follow-up in the referring hospitals were not considered. To assure sample’s representativity, baseline characteristics of included patients (Study population; n = 53) were compared to the baseline characteristics of all patients who received PMVR during the study period (n = 154). Clinical data of the patients were obtained from their medical records. This retrospective study was approved by the ethics committee of the Faculty of medicine, University RWTH Aachen. All research was performed in accordance with the relevant guidelines and regulations. The need of informed consent was waived by the ethics committee of the Faculty of medicine, University RWTH Aachen.

Prior to PMVR, all included patients had a symptomatic severe MR according to the recommendation of the European Association of cardiovascular imaging, i.e. EROA ≥ 40 mm^2^ or RVOL ≥ 60 ml/beat in primary MR and EROA ≥ 20 mm^2^ or RVOL ≥ 30 ml/beat in secondary MR^12^. All patients were discussed in the regular heart team meetings at our institution and were assessed to have a high surgical risk according to the logistic EuroScore^13^. The heart team included interventional and clinical cardiologists, imaging specialists, cardiovascular surgeons, and anesthesiologists. Medical records of all patients including transthoracic echocardiography (TTE), 3D TEE, coronary angiography and pulmonary function test were reviewed by the heart team. After visiting the patients at bedside, heart team recommended to proceed with PMVR in all patients.

### Percutaneous mitral valve repair

PMVR was performed using the MITRACLIP device (Abbott Vascular Structural Heart, Menlo Park, California). All procedures were conducted through experienced interventional cardiologists. The performance and steps of the procedure are described in detail elsewhere^3,4^. The intervention was carried out in general anesthesia and under guidance of fluoroscopy and 3D TEE.

### Echocardiographic examinations

All included patients received an echocardiographic follow-up with TTE and TEE at 6 months. Preprocedural and follow-up echocardiographic examination (TTE, TEE) were performed using a commercially available echocardiographic system (EPIQ 7, Philips Medical System, Andover, Massachusetts). Echocardiographic examinations were performed according to the recommendation of European and American society of echocardiography. Quantification of cardiac chambers were performed using TTE. Left ventricular dimensions were measured in the parasternal long axis. Left ventricle ejection fraction was assessed using Simpson’s method. Left and right atrium were assessed in the apical 4-chamber view. Right ventricular dimensions were measured in 4-chamber apical view at the base of the ventricle^14^. Echocardiographic parameters of the patients were obtained from their echocardiographic reports. Echocardiographic success of the procedure was defined by a reduction in MR severity to a maximally moderate residual regurgitation. Persistence of iASD was defined as presence of color doppler jet across the interatrial septum.

### Clinical follow-up at 6-months

An elective follow-up appointment was organized before discharge. Patients were evaluated clinically through interview and physical examination. Clinical outcome was evaluated based on improvement of heart failure symptoms represented by the mean reduction of NYHA dyspnea class. Clinical success of the procedure was defined as a reduction of NYHA class of at least one grade (Δ NYHA ≥ 1).

### Long term clinical follow-up

We contacted the patients, their families, or primary care-providers per telephone after a median time of 4.3 ± 1.5 years. Duration of long-term follow-up was defined as 2 years. Endpoints for long-term follow up were rehospitalization due to heart failure or mortality. Patients were asked about the need and eventually the time of rehospitalization due to heart failure in relation to the MitraClip procedure. Regarding patients who died during the follow up, date of death was obtained from their families or primary care providers.

### Statistical analysis

Continuous variables were expressed as mean ± standard deviation and binary variables were expressed as count (percentage). Baseline clinical and echocardiographic characteristics of included patients against all patients who received MITRACLIP during the study’s period were analyzed and compared using T-test for continuous- and chi-squared test for binary variables. According to the presence of iASD in the TEE examination at 6 months, patients were classified into two groups: iASD group vs. non-iASD group. Pre- and post-interventional clinical and echocardiographic parameters in each group (Intragroup comparison) were performed using T-test for paired samples for continuous variables and the Wilcoxon-signed rank test for ordinal variables (NYHA class, severity of MR and tricuspid regurgitation (TR). After that, we compared the mean reduction in NYHA dyspnea class and the reduction in MR severity between the two groups (Inter-group comparison) at 6 months to evaluate a possible impact of iASD on the outcome of the procedure. Rate of hospitalization and mortality at two-years follow-up were compared between the two groups. Kaplan–Meier curve for survival at long term follow-up (4.3 ± 1.5 years) was created. Uni- and multivariate logistic regression analysis was used to calculate the odds ratio for individual parameters to predict the persistent iASD at 6-months follow-up.

Statistical analyses were performed with SPSS version 25.0 (IBM Corp., Armonk, NY, USA). Statistical significance was awarded by p < 0.05.

## Results

53 (76 ± 9 years, 30 male) consecutive patients with symptomatic mitral regurgitation and high surgical risk who underwent PMVR were included in this study.

### Patients’ characteristics

Baseline clinical, echocardiographic, and procedural characteristics of study population are summarized (Table 1). Except for the presence of peripheral artery disease, there was no significant difference between included patients (n = 53) and all patients who received MITRACLIP during the study’s period (n = 154) (Table 2).

**Table 1:** Baseline characteristics of study population differentiating iASD and non-iASD group.

Variable	All patients N = 53	iASD group N = 33	Non-iASD group N = 20	P-Value
Age, year	76 ± 9	75.5 ± 9	76.2 ± 9	0.895
Female, n	23 (43)	19 (58)	4 (20)	**0.007**
**NYHA class, n (%)**				0.096
II	5 (9)	4 (12)	1 (5)	
III	27 (51)	13 (40)	14 (70)	
IV	21 (40)	16 (48)	5 (25)	
BMI, kg/m^2^	26 ± 6	26 ± 5	25 ± 4	0.525
Arterial hypertension, n (%)	40 (75)	25 (76)	15 (75)	0.906
Coronary artery disease, n (%)	38 (71)	23 (69)	15 (75)	0.410
COPD, n (%)	19 (35)	12 (36)	7 (35)	0.982
Chronic kidney disease, n (%)	33 (62)	19 (57)	14 (70)	0.308
Diabetes mellitus, n (%)	21 (39)	12 (36)	9 (45)	0.533
Peripheral artery disease, n (%)	10 (18)	7 (21)	3 (15)	0.613
Atrial fibrillation, n (%)	42 (79)	26 (78)	16 (80)	0.872
Cerebral artery disease, n (%)	7 (13)	5 (15)	2 (10)	0.622
Logistic Euro Score (%)	24 ± 12	25 ± 13	22 ± 11	0.639
Primary MR, n (%)	10 (19)	6 (18)	4 (20)	0.588
EROA, mm^2^	33 ± 11	33 ± 11	33 ± 13	0.990
RVOL, ml	55 ± 20	55 ± 20	55 ± 22	0.978
Left atrium area, cm^2^	29 ± 11	29 ± 13	29 ± 8	0.892
LVEDd, mm	56 ± 10	57 ± 10	53 ± 10	0.194
LVESd, mm	45 ± 12	46 ± 13	43 ± 12	0.319
EF, %	38 ± 15	35 ± 14	43 ± 15	**0.026**
RVEDd, mm	40 ± 6	39 ± 6	41 ± 5	0.139
RVSP, mmHg	43 ± 14	42 ± 11	37 ± 10	0.112
TAPSE, mm	15 ± 7	13 ± 7	16 ± 7	0.249
Right atrium area, cm^2^	23 ± 7	23 ± 8	23 ± 6	0.742
**Severity of TR, n (%)**				0.218
Mild	24 (45)	15 (46)	9 (45)	
Moderate	21 (40)	11 (33)	10 (50)	
Severe	8 (15)	7 (21)	1 (5)	
Number of clips	1.6 ± 0.5	1.6 ± 0.5	1.6 ± 0.7	0.836
Pmean across MV, mmHg	3.7 ± 1.6	3.7 ± 1.6	3.8 ± 1.7	0.988

*NYHA* New York Heart failure Association, *BMI* Body Mass Index, *COPD* Chronic Obstructive Pulmonary Disease, *MR* mitral regurgitation, *EROA* Effective regurgitation orifice area, *RVOL* Regurgitant volume, *LVEDd* Left Ventricle END Diastolic diameter, *LVESd* Left Ventricle End Systolic diameter, *EF* Ejection Fraction, *RVEDd* Right Ventricle End Diastolic diameter, *RVSP* Right Ventricle Pressure measured in echocardiography, *TAPSE* Tricuspid annular plane systolic excursion, *TR* Tricuspid Regurgitation.

Pmean: mean pressure across the mitral valve.

**Table 2:** Comparison of baseline characteristics between study population and all patients who received PMVR during study period.

Variable	All patients, n = 154	Study population, n = 53	p-value
Age, year	75 ± 9	76 ± 9	0.47
Female, n	54 (35)	23 (43)	0.29
**NYHA class, n (%)**			0.52
II	25 (16)	5 (9)	
III	71 (46)	27 (51)	
IV	58 (38)	21 (40)	
BMI, kg/m^2^	26 ± 5	26 ± 6	0.73
Arterial hypertension, n (%)	130 (85)	40 (75)	0.11
Coronary artery disease, n (%)	114 (74)	38 (71)	0.5
COPD, n (%)	71 (46)	19 (35)	0.32
Chronic kidney disease, n (%)	99 (64)	33 (62)	0.74
Diabetes mellitus, n (%)	56 (36)	21 (39)	0.69
Peripheral artery disease, n (%)	57 (37)	10 (18)	**0.014**
Atrial fibrillation, n (%)	110 (71)	42 (79)	0.32
Cerebral artery disease, n (%)	19 (12)	7 (13)	0.91
Logistic Euro Score (%)	27 ± 13	24 ± 12	0.26
Primary MR, n (%)	27 (17)	10 (19)	0.84
EROA, mm^2^	41 ± 13	33 ± 11	0.23
RVOL, ml	59 ± 24	55 ± 20	0.53
Left atrium area, cm^2^	30 ± 9	29 ± 11	0.25
LVEDd, mm	58 ± 9	56 ± 10	0.15
LVESd, mm	47 ± 12	45 ± 12	0.46
EF, %	38 ± 13	38 ± 15	0.83
RVEDd, mm	38 ± 6	40 ± 6	0.94
RVSP, mmHg	41 ± 12	43 ± 14	0.39
TAPSE, mm	16 ± 4	15 ± 7	0.32
Right atrium area, cm^2^	22 ± 6	23 ± 7	0.62
**Severity of TR, n (%)**			0.89
Mild	64 (43)	24 (45)	
Moderate	72 (45)	23 (44)	
Severe	18 (12)	6 (11)	
Number of clips	1.6 ± 0.6	1.6 ± 0.5	0.06
Pmean across MV, mmHg	3.6 ± 1.6	3.7 ± 1.6	0.91

*NYHA* New York Heart failure Association, *BMI* Body Mass Index, *COPD* Chronic Obstructive Pulmonary Disease, *MR* mitral regurgitation, *EROA* Effective regurgitation orifice area, *RVOL* Regurgitant volume, *LVEDd* Left Ventricle END Diastolic diameter, *LVESd* Left Ventricle End Systolic diameter, *EF* Ejection Fraction, *RVEDd* Right Ventricle End Diastolic diameter, *RVSP* Right Ventricle Pressure measured in echocardiography, *TAPSE* Tricuspid annular plane systolic excursion, *TR* Tricuspid Regurgitation.

Pmean: mean pressure across the mitral valve.

### Prevalence of persistent iASD

**Figure 1:**
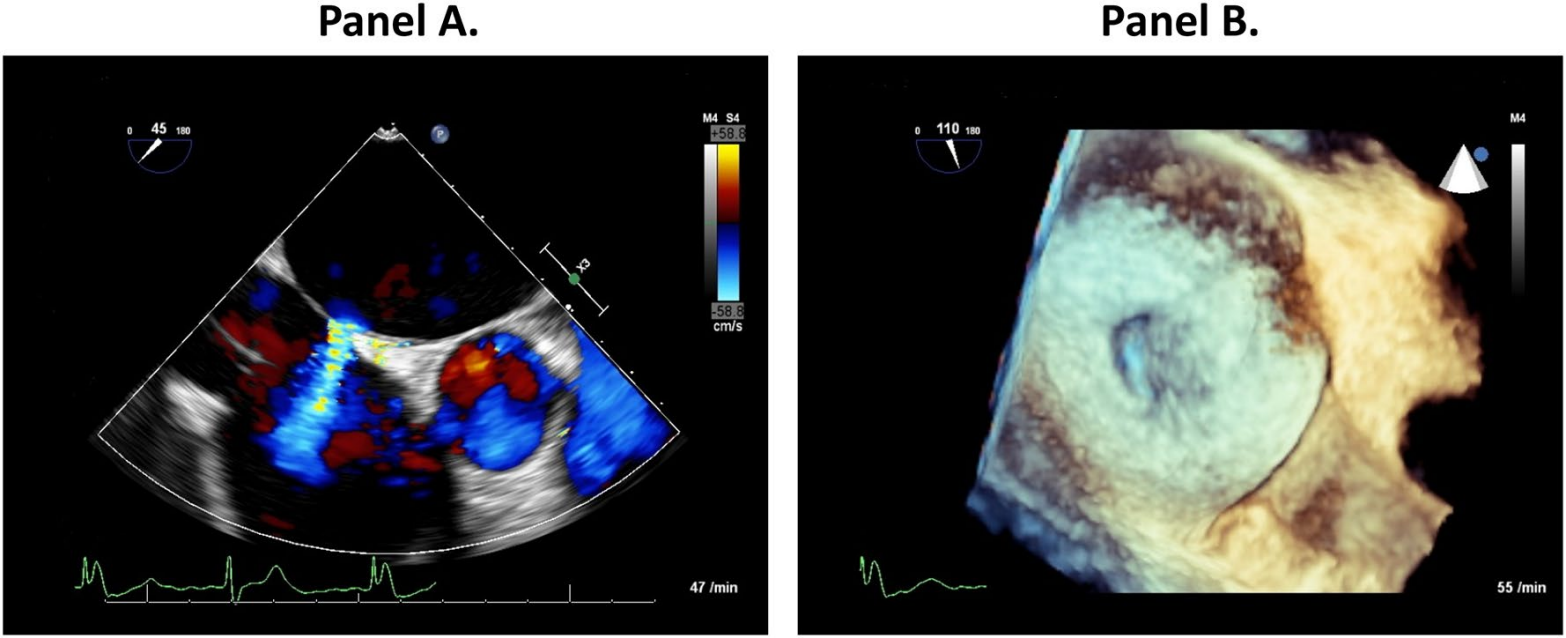
Iatrogenic atrial septal defect (iASD) at 6 months follow-up after MitraClip procedure. Panel (**A**) Transesophageal echocardiography with colour Doppler jet showing left to right shunting. Panel (**B**) 3D transesophageal echocardiography direct en-face imaging of the area of the iatrogenic atrial septal defect.

TEE at 6-months following MITRACLIP procedure revealed persistent iASD in 33 of 53 patients (62%) (Fig. 1). All shunts were unidirectional with blood flow through iASD directed from left into right atrium.

### Differentiating iASD group vs. non-iASD group

Other than gender, there were no significant intergroup differences in clinical characteristics between iASD group and non-iASD group. There were more female patients in the iASD group compared with non-iASD group (58% vs 20%, p = 0.007, respectively). Regarding echocardiographic parameters, baseline left ventricular ejection fraction (LV-EF) was lower in patients with persistent iASD compared with those without iASD (35 ± 14% vs. 43 ± 15%, p = 0.026, respectively). The procedure was completed in all patients with more than one clip being implanted in 58% of patients.

### 6-months clinical and echocardiographic follow-up

#### Patients with persistent iASD

At 6 months follow-up, there was a significant reduction in the severity of MR with 25 patients (76%) having a residual mild MR, six patients (18%) having a residual moderate MR and only two patients (6%) still having severe MR. Furthermore, a significant reduction of NYHA class from 3.3 ± 0.7 to 2.1 ± 0.8, p < 0.001 with a mean reduction (ΔNYHA) of 1.3 ± 1.0 was observed. The predefined clinical success was achieved in 25 of 33 patients (76%). However, there was a significant increase in basal right ventricular end diastolic diameter (RVEDd) from 39 ± 6 mm at baseline to 42 ± 8 mm, p < 0.001. There was no difference in right atrial area, right ventricular systolic function, represented by tricuspid annular plain systolic excursion (TAPSE) (13 ± 7 at baseline vs. 14 ± 7 mm at 6-months follow-up, p = 0.731) or right ventricular systolic pressure (RVSP) (42 ± 11 at baseline vs 40 ± 12 mmHg at 6-months follow-up, p = 0.735). There was no significant change in the severity of tricuspid regurgitation (TR) as well (Table 3). Closure of iASD was not performed in any of the patients.

**Table 3:** Clinical and echocardiographic characteristics at baseline and at 6-month follow-up in patients with and without persistent iASD and comparison of follow up between both groups.

Variable	iASD group			non-iASD group			p-value*
	Baseline	Follow-up	p-value	Baseline	Follow-up	p-value	
**NYHA class, n (%)**			** < 0.001**			**0.001**	
I	0 (0)	8 (24)		0 (0)	2 (10)		
II	4 (12)	14 (42)		1 (5)	11 (55)		
III	13 (40)	10 (31)		14 (70)	6 (30)		
IV	16 (48)	1(3)		5 (25)	1 (5)		
NYHA (mean)	3.3 ± 0.7	2.1 ± 0.8	** < 0.001**	3.2 ± 0.5	2.3 ± 0.7	**0.001**	0.444
Δ NYHA (mean)	1.3 ± 1			0.9 ± 1			0.171
Δ NYHA ≥ 1, n (%)	25 (76)			12 (60)			0.226
**Severity of MR, n (%)**			** < 0.001**			** < 0.001**	0.791
Mild	0 (0)	25 (76)		0 (0)	14 (70)		
Moderate	0 (0)	6 (18)		0 (0)	4 (20)		
Severe	33 (100)	2 (6)		20 (100)	2 (10)		
Left atrium area, cm^2^	29 ± 13	29 ± 13	0.912	29 ± 8	27 ± 8	0.003	0.496
LVEDd, mm	57 ± 10	58 ± 10	0.525	53 ± 10	54 ± 10	0.723	0.177
LVESd, mm	46 ± 13	49 ± 12	0.118	43 ± 12	42 ± 12	0.715	0.045
EF, %	35 ± 14	36 ± 13	0.066	43 ± 15	43 ± 13	1.0	0.078
RVEDd, mm	39 ± 6	42 ± 8	** < 0.001**	41 ± 5	39 ± 4	**0.002**	**0.047**
RVSP, mmHg	42 ± 11	40 ± 12	0.735	37 ± 10	35 ± 10	0.466	0.136
TAPSE, mm	13 ± 7	14 ± 7	0.731	16 ± 7	16 ± 8	0.803	0.176
Right atrium area, cm^2^	23 ± 8	23 ± 10	0.992	23 ± 6	20 ± 6	**0.014**	0.184
**Severity of TR**			0.744			0.096	0.191
Mild	15 (46)	16 (48)		9 (45)	14 (70)		
Moderate	11 (33)	10 (31)		10 (50)	5 (25)		
Severe	7 (21)	7 (21)		1 (5)	1 (5)		

*p-value for the comparison of outcome between the two groups at 6 months follow-up.

*NYHA* New York Heart failure Association, *Δ NYHA* change of NYHA class before and after the procedure, Δ NYHA ≥ 1, *n (%)* Number of patients who met the defined clinical success of the procedure, *MR* mitral regurgitation, *LVEDd* Left Ventricle END Diastolic diameter, *LVESd* Left Ventricle End Systolic diameter, *EF* Ejection Fraction, *RVEDd* Right Ventricle End Diastolic diameter, *RVSP* Right Ventricle Pressure measured in echocardiography, *TAPSE* Tricuspid annular plane systolic excursion, *TR* Tricuspid Regurgitation.

#### Patients without iASD

At 6-months follow-up, there was a significant reduction in the severity of MR with 14 patients (70%) having a residual mild MR, 4 patients (20%) having a residual moderate MR and only 2 patients (10%) still having severe MR. As well, there was a significant reduction of NYHA dyspnea class from 3.2 ± 0.5 to 2.3 ± 0.7, p = 0.001 with a mean reduction ΔNYHA of 0.9 ± 1. The predefined clinical success of MITRACLIP procedure was achieved in 12 of 20 patients (60%). Furthermore, there was a significant regression of right atrial and right ventricular dimensions (Table 3).

#### Comparison of clinical and echocardiographic outcome between iASD- and non-iASD group

Clinical outcome of the procedure was not significantly different between the two groups. There was no significant difference in the mean reduction of NYHA class between iASD and non- iASD group (1.3 ± 1 vs. 0.9 ± 1, p = 0.171, respectively). As well, the predefined clinical success of MITRACLIP procedure did not significantly differ between groups (76% of patients in iASD group vs. 60% of patients in non-iASD group, p = 0.226). However, there was a significant difference in RVEDd at 6 months between both groups (42 ± 8 mm in iASD group vs. 39 ± 4 mm in those without iASD, p = 0.047), whereas there was no significant difference in RVSP (40 ± 12 mmHg in iASD group vs. 35 ± 10 mmHg in non-iASD group, p = 0.136). Furthermore, RA area, TAPSE and severity of TR were still comparable between both groups (Table 3).

#### Two-years and long-term follow up

**Figure 2:**
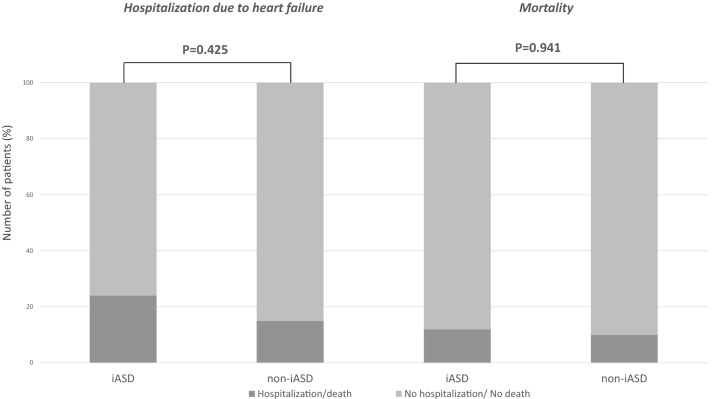
Comparison the rate of rehospitalization due to heart failure and mortality at 2 years follow-up between patients with and without iASD.

**Figure 3:**
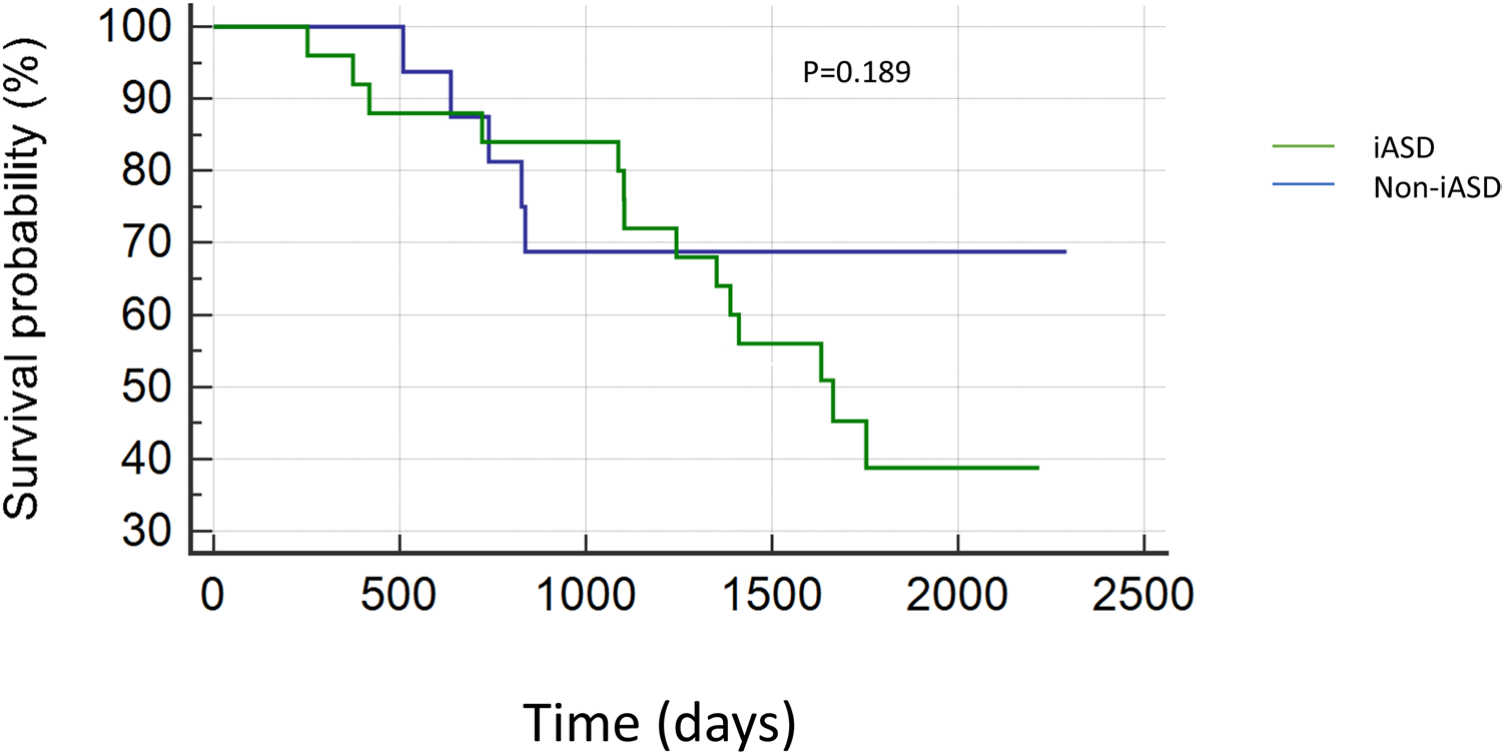
Kaplan Meier curve for survival in both groups at median follow-up of 4.3 ± 1.5 years.

During the predefined follow-up period of 2 years, rehospitalization due to decompensated heart failure occurred in 8 patients in iASD group (24%) and in 3 patients in non-iASD group (15%), p = 0.425. During the same follow-up period, 4 patients died in the iASD group (12%) and 2 patients (10%) died in non-iASD group, p = 0.941(Fig. 2). Kaplan–Meier curve for long term survival over a median follow-up of (4.3 ± 1.5 years) did not show significant difference in rate of survival between the two groups p = 0.189 (Fig. 3).

#### Predictors of persistent iASD at 6-months follow-up

Univariate logistic regression analysis of patients’ characteristics mentioned in Table 2 showed that female gender (OR = 5.4, 95% CI 1.48–19.82, p = 0.010) and reduced LV-EF (OR = 0.95, 95% CI 0.91–0.99, p = 0.031) were associated with persistent iASD at 6-months follow-up. In the multivariate analysis, female gender (OR = 5.5, 95% CI 1.43–21.42, p = 0.013) and reduced LVEF (OR = 0.95, 95% CI 0.91–0.99, p = 0.040) remained as independent predictors for persistent iASD at 6-months follow-up.

## Discussion

The main findings of this study were: (1) Incidence of persistent iASD at 6 months follow-up after MITRACLIP procedure was high and found to be 62%. (2) Persistence of iASD causes volume overload on the right heart without affecting its function or RVSP. (3) Persistent iASD had no impact on clinical outcome at 6 months or at the rate of rehospitalization or mortality at 2-years follow up. (4) Independent predictors for persistence iASD were female gender and reduced LVEF.

### Prevalence of iASD following MitraClip procedure

Over the last two decades, the number of transcatheter structural heart interventions through transseptal access has been markedly increased^15^. Use of transseptal access results in an iASD^16^. The incidence and impact of persistent iASD after PMVR on clinical and echocardiographic outcome were investigated in some studies^5–10^. However, the results were diverse and inconclusive^11^. Smith et al. reported the prevalence of persistent iASD in 27% of cases as detected by TTE at 6 months follow-up^5^. Ikenega et al. demonstrated in a retrospective study with 131 patients the presence of persistent iASD in 57% of cases at 1 month and in 18% at 12 months follow-up using TTE^6^. Saitaoh and coworkers reported in a retrospective study with 11 patients persistent iASD in 81% of cases at 1-month follow-up using TEE^7^. Schueler and colleagues detected persistent iASD in 50% of cases at 6-months follow-up using TEE^9^. These differences in the prevalence of iASD may be explained through the diverse duration of follow-up and the diverse imaging modalities which have been used in the above-mentioned studies. Our results support the findings of Schueler and colleagues, as we also found a high incidence of persistent iASD at 6 months follow-up using TEE.

### Predictors of persistent iASD

Predictors for persistent iASD following MITRACLIP procedure were previously described in some studies. Smith and coworkers showed that patients with persistent iASD had less reduction of MR and a trend towards larger left atrial (LA) volumes^5^. Ilkenaga and colleagues showed that mean LA pressure at 1-month follow-up was associated significantly with persistent iASD at 12 months follow-up in patients after MITRACLIP procedure^6^. In the present study, LVEF at baseline was significantly lower in patients with persistent iASD compared to those without persistent iASD. As described by Kainuma et al.^17^, reduced LVEF at baseline and at discharge was associated with elevated pulmonary capillary wedge pressure in 46 patients with surgical mitral valve repair, suggesting that reduced LVEF in the present study may have been associated with elevated mean LA pressure, which was the main mechanism for persistent iASD described by Ilkinaga and coworkers^6^. Furthermore, female gender was in the present study an independent predictor for persistent iASD. Gender differences in many clinical and genetic aspects of cardiovascular disease are well described^18–20^. Till now, there is no physiologic or anatomic explanation of this finding and it should be further studied. Nonetheless, this finding may have been spurious due to small sample size.

### Impact of persistent iASD on clinical outcome

Available data on the impact of persistent iASD on clinical outcome after MITRACLIP procedure are controversial. Ikenaga and colleagues reported no significant differences in right heart dimensions and estimated systolic pulmonary artery pressure between patients with and without persistent iASD at 12 months follow-up. They also reported no differences in NYHA class and brain natriuretic peptide level between both groups^6^. On the other side, Toyama and coworkers reported that persistent iASD at 12 months follow-up was associated with right heart enlargement, increased severity of TR and increased rate of re-hospitalization due to heart failure^8^. In a prospective study of 66 patients, Schueler and colleagues demonstrated in a that persistent iASD at 6 months follow-up was associated with a worse clinical outcome and with an increased mortality^9^. Controversially, in a prospective study of 80 patients, Larz et al. showed no significant improvement in clinical outcome in patients who received an interventional closure of persistent iASD after MitraClip compared to conservative therapy^10^. However, and as mentioned by both authors, this data must be interpreted with caution due to relatively short duration of follow up. Some authors reported individual cases of interventional closure of iASD after PMVR whether due to acute right heart failure with right-to-left shunt or due to progressive increase in iASD diameter with progressive dyspnea and recurrent hospitalization^21,22^. Our findings support the results of Larz et al., showing that persistent iASD did not affect clinical outcome after the procedure, as mean reduction of NYHA dyspnea class did not differ between patients with and without iASD. Nonetheless, persistent iASD led to a mild volume overload on the right ventricle leading to its dilation. Fortunately, this was not accompanied with deterioration in the right ventricular systolic function represented by TAPSE or with an increase in the estimated pulmonary pressure resembled by RVSp, therefore no closure of iASD was considered in our study population. The possible negative symptomatic effect of volume overload on the right ventricle, might have been encountered by a positive effect of volume- and pressure relief delivered to left atria. A previous study at our institution performed an invasive measurement of mean LA pressure in 28 patients with iASD after MITRACLIP and showed a reduction in LA pressure from 17 ± 8 mm Hg to 15 ± 8 mm Hg after withdrawal of the guiding catheter, which means that iASD resulted in an immediate volume and pressure relief in LA^23^. Correspondingly, Feldman et al. could demonstrate in a randomized, multicenter study (REDUCE LAP-HF 1) that iASD reduced pulmonary capillary wedge pressure during exercise in patients with heart failure and LVEF > 40%^24^. Also, Del Trigo and coworkers reported that unidirectional left to right interatrial shunting for treatment of patient with heart failure with reduced LVEF was associated with early beneficial clinical hemodynamic outcomes^25^.

Ikenaga and colleagues did not report any event of ischemic stroke or other types of paradoxical embolism during the follow-up period as well^6^^.^ Toyama and colleagues reported that the incidence of stroke was comparable between both groups during the follow-up period of 1 year^8^. However, in our study, there was no ischemic stroke or paradoxical embolism during the follow-up period.

Conclusively, our study did not show any significant difference between patients with and without iASD after PMVR and did not demonstrate any relevant effect of persistent iASD on the outcome of the procedure or neither at 6 months- nor at 2 years follow-up.

## Limitations

Although this study adds further information to the knowledge about iASD after MITRACLIP procedure, we acknowledge that it is mainly limited by its retrospective design. Although diameter of atrial septal defect does not represent solely an indication for intervention, no measurement of iASD diameter was performed in our study. Despite being the first study to report about effect of iASD at long-term follow-up, this study is limited by the small number of patients. In addition, this investigation suffers the usual shortcomings of a single center study.

## Conclusions

Incidence of persistent iASD after MITRACLIP procedure is markedly high. Female gender and reduced LVEF were independent predictors for persistence of iASD. Despite the increase in right ventricular size, patients with iASD were not clinically compromised compared with those without persistent iASD at 6 months follow-up. Persistent iASD did not lead to increased rate of rehospitalization or increased mortality at 2 years follow-up.
